# Meetings of Societies

**Published:** 1936

**Authors:** 


### Meetings of Societies.

THE seventh meeting of the session was held on Wednesday, 8th April, at the University. The President, Mr. A. E. Iles, was in the Chair and fifty-four members were present.

Dr. A. L. Taylor showed pathological specimens, and Dr. Bergin demonstrated skiagrams.

Then followed a discussion on "**A Treatment for Sciatica.**" The anatomical aspect was introduced by Professor Whitnall, who was followed by Dr. Parkes.

The surgical aspect was discussed by Professor Short, and in the general discussion which ensued the following gentlemen took part: Dr. Carleton, Dr. Richard Clarke, Dr. Orr-Ewing, Dr. Glenny, Professor Nixon and Mr. Handley Howell.

---

The eighth meeting of the session was held on Wednesday, 13th May, at the University. The President, Mr. A. E. Iles, was in the Chair and eighty-six members were present.

Dr. Price showed pathological specimens.

The President then introduced Mr. Wilfrid Sheldon, who delivered his lecture on "**The Interpretation of Abdominal Pain in Childhood.**"

A brief discussion followed, and Dr. Carleton proposed and Professor Perry seconded the vote of thanks, which was carried, showing the great appreciation of a very admirable lecture.

